# Predictive Model for Overall Survival and Cancer-Specific Survival in Patients with Esophageal Adenocarcinoma

**DOI:** 10.1155/2021/4138575

**Published:** 2021-09-14

**Authors:** He Huang, Weiyue Fang, Ying Lin, Zhanzhong Zheng, Zefan Wang, Xiangfen Chen, Kang Yu, Guangrong Lu

**Affiliations:** ^1^Department of Hematology and Oncology, The Second Affiliated Hospital and Yuying Children's Hospital of Wenzhou Medical University, Wenzhou, Zhejiang, China; ^2^Wenzhou Medical University, Wenzhou, Zhejiang, China; ^3^Department of Hematology, The First Affiliated Hospital of Wenzhou Medical University, Wenzhou 325027, Zhejiang, China; ^4^Department of Gastroenterology, The Second Affiliated Hospital and Yuying Children's Hospital of Wenzhou Medical University, Wenzhou, China

## Abstract

**Objective:**

Recent years, there has been a rapid increase in the incidence of esophageal adenocarcinoma (EAC), while the prognosis for patients diagnosed remains poor and has slightly improved.

**Methods:**

We extracted 6,466 cases with detailed demographical characteristics including age at diagnosis, sex, ethnicity, marital status, and clinical features, involving tumor grade and stage at diagnosis and treatment modalities (radiation therapy, chemotherapy, and surgery) from the Surveillance, Epidemiology, and End Results (SEER) (1975–2017) dataset. They were further randomly divided into the training and validating cohorts. Univariate and multivariate Cox analyses were conducted to determine significant variables for construction of nomogram. The predictive power of the model was then assessed by Harrell concordance index (C-index) and the area under the curve (AUC) of the receiver operating characteristic (ROC) curve.

**Results:**

Multivariate analysis revealed that age, marital status, insurance, tumor grade, TNM stage, surgery, and chemotherapy all showed a significant association with overall survival (OS) and cancer-specific survival (CSS). These characteristics were employed to build a nomogram. Particularly, the discrimination of nomogram for OS and CSS prediction in the training set were excellent (C-index = 0.762, 95% CI: 0.754–0.770 and C-index = 0.774, 95% CI: 0.766–0.782). The AUC of the nomogram for predicting 2- and 5-year OS was 0.834 and 0.853 and CSS was 0.844 and 0.866. Similar results were observed in the internal validation set.

**Conclusion:**

We have successfully established a novel nomogram for predicting OS and CSS in EAC patients with good accuracy, which can help clinicians predict the survival of individual patient survival and provide optimal treatment strategies.

## 1. Introduction

The estimated incidence of esophageal adenocarcinoma (EAC) in the United States was 17,650 in 2019 [1], and the incidence rate of EAC has surpassed that of esophageal squamous cell carcinoma (ESCC), becoming the main histologic type of esophageal cancer in the West [2–4]. Despite a significant increase in its incidence, the 5-year survival for EAC has improved only marginally, from 9% in the 1970s to 22% in 2009 [5]. Prior epidemiological studies have demonstrated associations between EAC and family history, smoking, older age, male gender, central obesity, gastroesophageal reflux disease (GERD), and Barrett's esophagus (BE) [6]. However, it is evident that the prognostic system derived from the Kaplan–Meier estimator becomes less relevant over time after diagnosis [7, 8], alarming a need for an improved predictive survival system for EAC patients.

According to the population-based Surveillance, Epidemiology, and End Results (SEER), multinomograms were developed based on a multivariate regression model for esophageal cancer (EC) [9, 10]. Currently, neoadjuvant chemoradiation followed by esophagectomy (trimodality therapy) is the standard treatment of locally advanced esophageal carcinoma [11], but a significant proportion of patients relapse and die after treatment. Despite several prognostic evaluations assessed trimodality therapy or pharmaceuticals treatments (proton pump inhibitors (PPIs), statins, nonsteroidal anti-inflammatory drugs (NSAIDs) and metformin) impacting the outcomes [12, 13], an ideal prognostic model with the value of accuracy and applicability for EAC needs to be set.

In this study, we developed a nomogram with a multivariate Cox proportional hazards regression model that incorporates comprehensive demographic and baseline clinical variables, including age, race, insurance and marital status, tumor grade, primary site, clinical stage, chemotherapy, surgery, and radiotherapy strategy. Using scaled line segments, various forecast indicators were listed and scored, and we developed and validated a new model predicting the overall survival (OS) and cancer-specific survival (CSS) for EAC patients. And the Harrell C-index and the area under the curve (AUC) of the receiver operating characteristic (ROC) curve used to indicate the performance of the nomogram were excellent. Thus, we believed this established novel nomogram for patients with EAC could assist clinicians in predicting the survival of individual patient.

## 2. Methods

### 2.1. Patients

**Figure 1:**
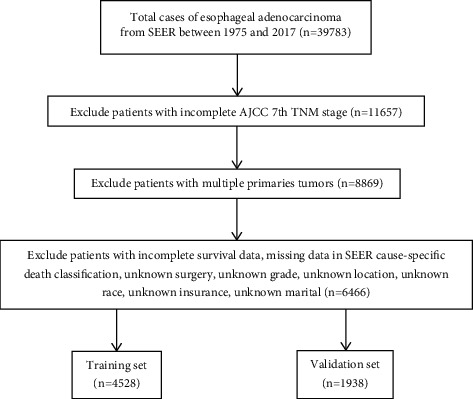
Flow diagram of EAC patients with training and validation cohorts.

A total of 39,783 EAC patients (between 1975 and 2017) were identified from the SEER registry database of the National Cancer Institute using SEER^*∗*^Stat software (version. 8.3.5), which covers about 28% of the US population and contains a large amount of evidence-based medical information [14]. Patients with the incomplete 7th edition of the American Joint Committee on Cancer (AJCC) Tumor-Node-Metastasis (TNM) staging system were excluded. Then, the patients with multiple primaries tumors were further excluded. In addition, patients with incomplete survival data, missing data in SEER cause-specific death classification, unknown surgery, unknown grade, unknown location, unknown race, unknown insurance, and unknown marital status were also excluded from the study. Finally, 6,466 cases enrolled were randomly assigned into the training set (4,528) and validation set (1,938) (Figure 1). Because all of the data used in this study were obtained from the SEER database with a publicly available method, no local ethical approval or declaration was required for this study. All data used in this study are publicly available (https://seer.cancer.gov/).

### 2.2. Construction and Validation of the Nomogram

The data of training cohort was used to establish the nomogram. The endpoint OS and CSS were measured from the date of first diagnosis to the date of any cause of death. Survival was estimated using the Kaplan–Meier method and Cox regression analysis. Univariate and multivariate analyses were performed to determine independent prognostic variables. Then, nomograms to predict the 2- and 5-year OS and CSS rates were constructed using the results of the multivariate analysis showing significance. The discriminatory performance of the nomograms was assessed by C-index and AUC. Calibration curves were created using the marginal estimation and the average prediction probability of the model. Furthermore, the nomograms were also compared to the AJCC 7th TNM stage in terms of C-index and AUC.

### 2.3. Statistical Analysis

Participant demographics were compared using the *X*^2^ test. All the statistical analyses were performed using R version 3.4.2 software (the R Foundation for Statistical Computing, Vienna, Austria. http://http://www.r-project.org). A two tailed *p* < 0.05 was considered statistically significant.

## 3. Results

### 3.1. Patient Characteristics

The demographic and clinical variables are listed in Table 1. Patients were grouped into >60 years (*n* = 1,972) and ≥60 years (*n* = 4,494) based on the age at diagnosis. There were 788 females (12.2%) and 5,678 males (87.8%), 167 Blacks (2.6%), 6,129 Whites (94.8%), 170 other racial people (2.6%), 4,062 married (62.8%), 2,404 unmarried (37.2%), 5,591 insured (86.5%), 704 with any Medicaid (10.9%), and 171 uninsured (2.6%), respectively. At the time of diagnosis, there were 432 grade I patients (6.7%), 2,647 grade II patients (40.9%), 3,310 grade III patients (51.2%), and 77 grade IV patients (1.2%). Most patients had the primary lesion at lower third esophagus (86.2%), followed middle third (8.4%). According to the AJCC TNM staging system, there were 1,053 stage I patients (16.3%), 1,045 stage II patients (16.2%), 1,845 stage III patients (28.5%), and 2,523 stage IV patients (39%). The majority of cases experienced surgery (65.4%), chemotherapy (70.3%), and radiotherapy (58.6%). In general, patients randomized into two cohorts shared similar clinical characteristics.

### 3.2. Patient Prognosis Analysis in the Training Cohort

The univariate and multivariate analyses in training cohort are listed in Table 2, and the values of multivariate were further assessed in condition of the *p* < 0.200 in the univariate analysis in terms of OS and CSS. Even male predominance in incidence is stronger than female as reported [15, 16], and there was negative discrepancy here. In univariate models for OS, age, race, marital status, insurance, tumor differentiation grade, primary site, tumor staging, surgery, chemotherapy, and radiation therapy (overall *p* < 0.05) were significantly associated with OS. In the multivariable age groups above 60 years (hazard ratio (HR) = 1.198, 1.567, 2.212; 95%), marital status (*p*=0.004), insurance (overall *p* < 0.005), poor tumor differentiation grade (grade III: HR = 1.455, 95% CI: 1.028–2.059, *p*=0.034; grade IV: HR = 1.558, 95% CI: 1.327–1.829, *p* ≤ 0.001), tumor staging (overall *p* ≤ 0.001), surgery, and chemotherapy (overall *p* ≤ 0.001) were independent predictors for OS. In the univariate and multivariate analyses of CSS, the parameters significantly associated with survival were consistent with the items of OS. In particular, radiotherapy did not impact OS or CSS of EAC patients with *p* values 0.354 and 0.289, respectively.

### 3.3. Nomograms for Predicting OS and CSS of EAC Patients

**Figure 2:**
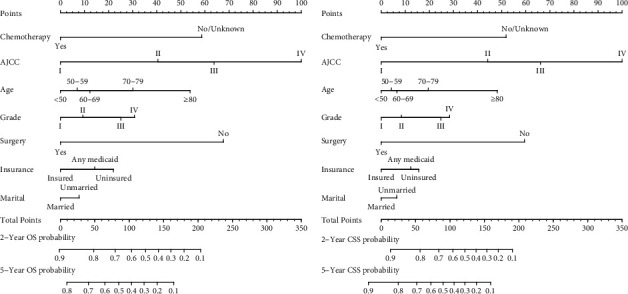
Nomograms predicting 2- and 5-year OS (a) and CSS (b) of patients with EAC.

**Figure 3:**
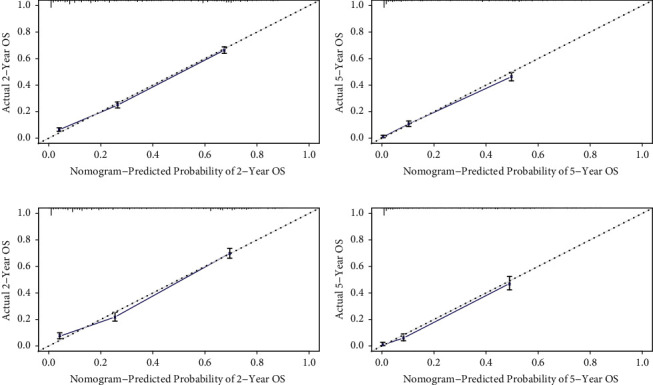
Calibration plots of the nomogram for 2- and 5-year OS prediction of the training cohort (a)–(b) and internal validation cohort (c)–(d).

**Figure 4:**
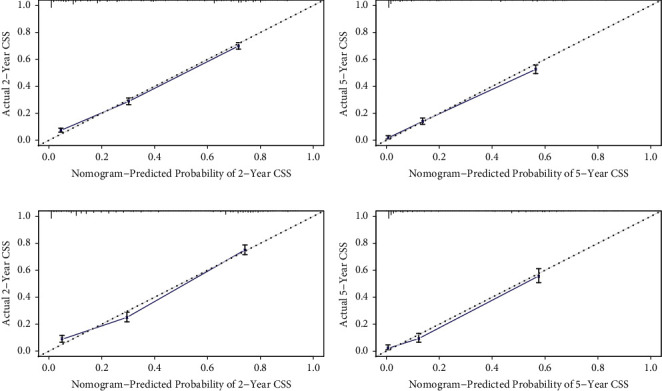
Calibration plots of the nomogram for 2- and 5-year CSS prediction of the training cohort (a)–(b) and internal validation cohort (c)–(d).

The nomograms based on the multivariate Cox regression models were developed to estimate 2-year and 5-year OS probabilities and CSS probabilities (Figure 2). By adding up the scores for each selected variable, a patient's probability of individual survival can be easily calculated, and the performance of the nomograms was assessed by calculating Harrell's C-index. The OS and CSS were better for patients under the age of 60, patients with comparative better tumor differentiation and early stages, patients insured and married, and patients received surgery or chemotherapy. The C-index for the nomogram to predict OS was 0.762 (95% CI: 0.754–0.770) for the training cohort and 0.770 (95% CI: 0.758–0.782) for the validation cohort. And nomogram accuracy for CSS prediction was observed with a C-index of 0.774 (95% CI: 0.766–0.782) for the training cohort and 0.783 (95% CI: 0.770–0.797) for the validation cohort. The nomogram for OS and CSS prediction demonstrated relatively good accuracy comparing to AJJC 7th TNM stage (Table 3). Then, calibration plots of 2- and 5-year OS probabilities confirmed optimal agreement between the nomogram-predicted survival and actual observations in both the training and internal validation sets (Figure 3), and so were CSS probabilities (Figure 4).

**Figure 5:**
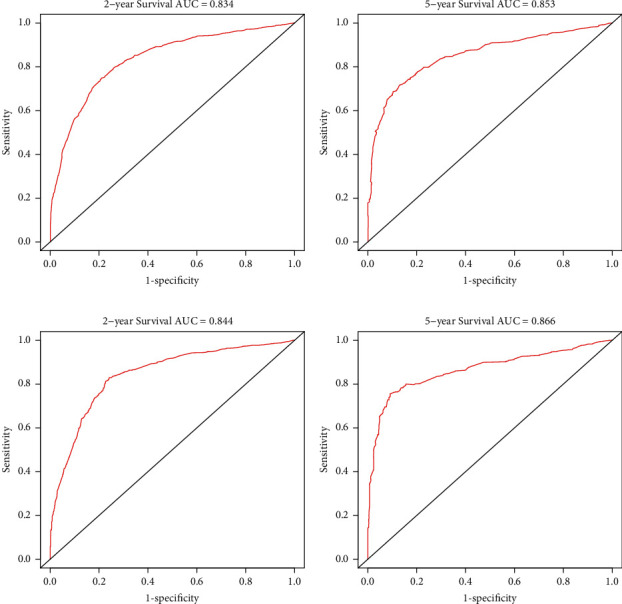
The ROC curves of the nomograms for 2- and 5-year OS prediction of the training cohort (a)-(b) and internal validation cohort (c)-(d).

**Figure 6:**
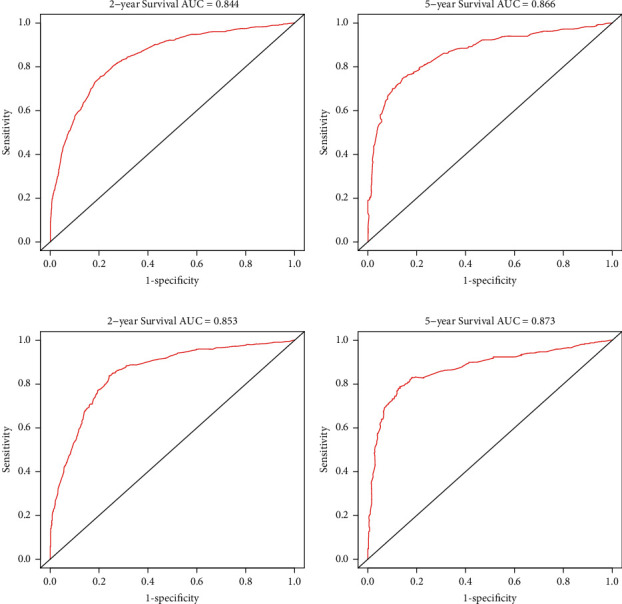
The ROC curves of the nomograms for 2- and 5-year CSS prediction of the training cohort (a)-(b) and internal validation cohort (c)-(d).

Additionally, AUC values of the ROC for the training cohort were 0.834 and 0.853 for the projected 2- and 5-year OS and 0.844 and 0.866 for the projected 2- and 5-year CSS, respectively. For the validation cohort, the AUC values of the nomogram for predicting the 2- and 5-year OS rates were 0.844 and 0.866 and 0.853 and 0.873 for CSS, respectively (Figures 5 and 6). So higher AUC values were observed for the nomogram comparing to the items of AJCC 7th TNM stage (Table 4).

## 4. Discussion

A previous study using the SEER 1973–2009 dataset reported that the overall 5-year survival rate was 9–22% in all EC patients [17]. Furthermore, the United States Cancer Statistics in 2018 reported that the 5-year overall relative survival of EC was 19% (2008 to 2014), and a hospital-based pooled analysis in China reported that the 5-year overall survival was around 40%, with an increase over time from 2000 to 2018 [18, 19]. Overall, the overall prognosis in EC is poor. Over the past 30 years, the incidence of EAC rapidly increased and had surpassed that of ESCC in a number of Western countries, including the United Kingdom (UK), the Netherlands, Ireland, New Zealand, the United States (US), Australia, Denmark, Canada, and Sweden [3, 20, 21]. With the steady increase in the number of EAC, there is a growing need for accurate estimates of disease outcomes. Using the rich data sources, the SEER-Medicare population, we identified 6,466 patients diagnosed with EAC between 1975 and 2017, which allowed for reliable analyses of subgroups and trends in survival after diagnosis. Furthermore, excellent predictive power of nomograms was confirmed by the higher C-index and AUC value comparatively both in the training and validation sets than the AJCC 7th TNM stage system.

In this study, we constructed well-calibrated prognostic nomograms to predict OS and CSS in patients with EAC. Consisting with prior research studies, predictive parameters including age, marital status, insurance, tumor differentiation, and TNM stage were associated with OS and CSS [12, 22–25]. Patients over 60 years of age, from a family relatively lack of care and support, with the poor tumor differentiation and in advanced stage had the worst prognosis. Interestingly, ethnic disparities and primary site that show independent prognostic factors in ESCC patients [25, 26] were not significant values for OS and CSS in EAC patients. That may be need further evidences to confirm the value of these parameters.

Surgery is the primary treatment for EC. Even EAC patients who received surgery just account for 34.6% (including endoscopic therapy, esophagectomy, with gastrectomy, and combination); our data showed that the OS and CSS of patients who underwent surgery were significantly longer than those who had no surgery. To our knowledge, patients with EAC more frequently received chemotherapy than patients with ESCC. Of note, ∼70.3% of patients experienced chemotherapy in our study, and chemotherapy also was an independent prognostic factor. Conversely, patients with ESCC were more likely to receive radiation therapy [10]. ∼58.6% of EAC patients here received radiotherapy, but suggested no significant association with prognosis. Radiotherapy plays a crucial role in the treatment of EC and almost was carried out before or after surgery. Our findings strengthen the previous study that showed no improvement in OS and CSS in stages I–III patients who received single or combined radiotherapy before and after surgery, compared with patients who did not experience radiotherapy [25]. They required further evidence-based data to learn.

The overall prognosis for patients has been markedly improved because of the awareness and surveillance of individual with Barrett's esophagus (BE), more accurate selection of patients for curative treatment, better surgical and perioperative therapy, and the addition of neoadjuvant chemotherapy or chemoradiotherapy for localized [5, 6, 27]. The postoperative mortality and complication rates of the disease are much higher compared to endoscopic therapy [28, 29], and EC patients with stages I–III underwent endoscopic therapy had the association with the best outcome amongst all the surgical methods, including esophagectomy and esophagectomy with gastrectomy [25]. Therefore, endoscopy, early screening of certain high-risk individuals to detect premalignant lesions, even further the option for treatment, is also a very important tool to guide the treatment and assessment of prognosis. This retrospective remained several considerable limitations. First, the inherent selection bias was inevitable. Second, this report based on the majority population was Whites. Due to the distribution of the EC, ESCC remains the most frequent histological type in Asian, from northern Iran, east to China, and north to Russia [30, 31], but since EAC is extremely rare in China, we lack data from real-world studies to check it. Furthermore, endoscopic therapy currently is prevalent in clinic, whereas this study missed subgroup data involved. Then, the etiology of EAC, including obesity [32], *Helicobacter pylori* infection [33, 34], tobacco smoking [32, 35], alcohol consumption, dietary factor [36], medication [13, 37], and genetic factor [38, 39], definitely impact the patients' survival, but were not involved here due to information incomplete. Although the tumor response to neoadjuvant chemotherapy or chemoradiotherapy is another important prognostic factor [27, 40], there are currently no known biomarkers or diagnostic modalities that can reliably predict a patient's response to neoadjuvant chemoradiation. Unfortunately, the addition of neoadjuvant chemotherapy or chemoradiotherapy for localized EAC was not discussed. Finally, the SEER-Medicare population research screened EAC cases from 1975 to 2017; there were considerable varies uncontrolled. Even the database provided the number of patients who received chemotherapy, radiotherapy, or surgery alone or in combination (Supplemental Table 1); we did not do more clarification about treatment strategies. The data available for analysis will be significantly reduced if we further group patients based on the time relationship between chemoradiotherapy and surgery (preoperative or postoperative). Correspondingly, the time association of most patients in the surgical group with chemoradiotherapy is unknown in the SEER database here, and the number of cases with detailed sequence is relatively significantly small. In general, we regret this confusion without more clarification. We believe the evidence will be further confirmed in future real-world studies. Ultimately, prospective multicentre studies are needed to validate and utilization this predictive nomogram.

## 5. Conclusion

We assessed a large number of cases and incorporated clinical information to construct and validate a universally applicable EAC prediction model that performed better C-index and AUC than the traditional TNM staging system. This nomogram can forecast the dynamic and personalized OS and CSS of patients during follow-up after diagnosis. Age, marital status, insurance, tumor grade, TNM stage, surgery, and chemotherapy were significant independent predictors of OS and CSS. EAC patients can benefit from this nomogram and accept more aggressive posttherapy surveillance, and clinicians can be guided to select treatment plans.

## Figures and Tables

**Table 1:** Patient characteristics.

Variables	Total (*n* = 6466)	Training cohort (*n* = 4528)	Validation cohort (*n* = 1938)	*p*
Age (year)				0.811
<60	1972 (30.5)	1385 (30.6)	587 (30.3)	
≥60	4494 (69.5)	3143 (69.4)	1351 (69.7)	
Sex				0.497
Female	788 (12.2)	560 (12.4)	228 (11.8)	
Male	5678 (87.8)	3968 (87.6)	1710 (88.2)	
Race				0.365
Black	167 (2.6)	109 (2.4)	58 (3.0)	
White	6129 (94.8)	4300 (95.0)	1829 (94.4)	
Others	170 (2.6)	119 (2.6)	51 (2.6)	
Marital status				0.353
Married	4062 (62.8)	2828 (62.5)	1234 (63.7)	
Unmarried	2404 (37.2)	1700 (37.5)	704 (36.3)	
Insurance				0.139
Insured	5591 (86.5)	3893 (86.0)	1698 (87.6)	
Any Medicaid	704 (10.9)	506 (11.1)	198 (10.2)	
Uninsured	171 (2.6)	129 (2.9)	42 (2.2)	
Grade				0.484
I	432 (6.7)	315 (7.0)	117 (6.0)	
II	2647 (40.9)	1835 (40.5)	812 (41.9)	
III	3310 (51.2)	2323 (51.3)	987 (50.9)	
IV	77 (1.2)	55 (1.2)	22 (1.2)	
Primary site				0.246
Upper third	67 (1.1)	46 (1.0)	21 (1.1)	
Middle third	545 (8.4)	380 (8.4)	165 (8.5)	
Lower third	5573 (86.2)	3890 (85.9)	1683 (86.8)	
Overlapping lesion	281 (4.3)	212 (4.7)	69 (3.6)	
AJCC 7th TNM stage				0.384
I	1053 (16.3)	722 (15.9)	331 (17.1)	
II	1045 (16.2)	724 (16.0)	321 (16.6)	
III	1845 (28.5)	1286 (28.4)	559 (28.8)	
IV	2523 (39.0)	1796 (39.7)	727 (37.5)	
Surgery				0.440
No	4229 (65.4)	2975 (65.7)	1254 (64.7)	
Yes	2237 (34.6)	1553 (34.3)	684 (35.3)	
Chemotherapy				0.365
No/unknown	1921 (29.7)	1330 (29.4)	591 (30.5)	
Yes	4545 (70.3)	3198 (70.6)	1347 (69.5)	
Radiation				0.583
No/unknown	2679 (41.4)	1886 (41.7)	793 (40.9)	
Yes	3787 (58.6)	2642 (58.3)	1145 (59.1)	

*Note.* If *t* ≥ 5, Pearson' *X*^2^ test; if 1≤*t*<5, the continuity correction *X*^2^ test. Grade I, high differentiated; II, moderate differentiated; III, poor differentiated; IV, undifferentiated. Unmarried includes single, divorced, and widowed.

**Table 2:** Univariate and multivariate analyses of survival in EAC patients.

Variables	Overall survival				Cancer-specific survival			
	Univariate analysis		Multivariate analysis		Univariate analysis		Multivariate analysis	
	Log rank *X*^2^	*p*	HR (95% CI)	*p*	Log rank *X*^2^	*p*	HR (95% CI)	*p*
Sex	0.497	0.481			0.757	0.384		
Female								
Male								
Age (years)	150.747	≤0.001		≤0.001	119.054	≤0.001		≤0.001
<50			Reference				Reference	
50–59			1.105 (0.962–1.269)	0.159			1.071 (0.930–1.235)	0.341
60–69			1.198 (1.053–1.362)	0.006			1.118 (0.980–1.275)	0.097
70–79			1.567 (1.336–1.837)	≤0.001			1.406 (1.191–1.660)	≤0.001
≥80			2.212 (1.641–2.982)	≤0.001			2.271 (1.675–3.081)	≤0.001
Race	6.397	0.041		0.816	6.614	0.037		0.809
Black			Reference				Reference	
White			1.006 (0.816–1.240)	0.953			1.011 (0.813–1.256)	0.924
Others			0.938 (0.696–1.263)	0.672			0.938 (0.687–1.279)	0.684
Marital status	38.416	≤0.001			34.036	≤0.001		
Married			Reference				Reference	
Unmarried			1.113 (1.035–1.197)	0.004			1.107 (1.026–1.195)	0.009
Insurance	57.232	≤0.001		≤0.001	53.570	≤0.001		≤0.001
Insured			Reference				Reference	
Any Medicaid			1.229 (1.102–1.371)	≤0.001			1.228 (1.096–1.376)	≤0.001
Uninsured			1.393 (1.146–1.693)	0.001			1.314 (1.069–1.615)	0.009
Grade	215.503	≤0.001		≤0.001	230.817	≤0.001		≤0.001
I			Reference				Reference	
II			1.148 (0.977–1.349)	0.094			1.155 (0.970–1.376)	0.106
III			1.455 (1.028–2.059)	0.034			1.531 (1.064–2.230)	0.022
IV			1.558 (1.327–1.829)	≤0.001			1.606 (1.351–1.910)	≤0.001
Primary site	18.631	≤0.001		0.280	20.771	≤0.001		0.229
Upper third			Reference				Reference	
Middle third			1.151 (0.822–1.612)	0.412			1.113 (0.784–1.582)	0.549
Lower third			1.101 (0.801–1.514)	0.552			1.057 (0.759–1.472)	0.743
Overlapping lesion			1.263 (0.891–1.792)	0.190			1.237 (0.860–1.778)	0.252
AJCC TNM stage (7th)	1397.996	≤0.001		≤0.001	1508.416	≤0.001		≤0.001
I			Reference				Reference	
II			1.823 (1.559–2.132)	≤0.001			2.147 (1.802–2.558)	≤0.001
III			2.567 (2.224–2.963)	≤0.001			3.114 (2.651–3.658)	≤0.001
IV			4.243 (3.670–4.904)	≤0.001			5.371 (4.565–6.319)	≤0.001
Surgery	1334.023	≤0.001			1302.575	≤0.001		
No			Reference				Reference	
Yes			0.366 (0.331–0.405)	≤0.001			0.360 (0.323–0.401)	≤0.001
Chemotherapy	70.954	≤0.001			52.342	≤0.001		
No/unknown			Reference				Reference	
Yes			0.443 (0.405–0.483)	≤0.001			0.434 (0.396–0.476)	≤0.001
Radiation	70.727	≤0.001			69.422	≤0.001		
No/unknown			Reference				Reference	
Yes			0.963 (0.890–1.042)	0.354			0.957 (0.881–1.038)	0.289

*Note.* Univariate analysis, Kaplan–Meier analysis; multivariate analysis, Cox regression analysis; HR, hazard ratio.

**Table 3:** C-index for the nomogram and TNM stage systems in patients with EAC.

Survival		Training cohort	*p*	Internal validation cohort	*p*
OS	Nomogram	0.762 (0.754–0.770)	<0.001	0.770 (0.758–0.782)	<0.001
	7th TNM stage	0.675 (0.665–0.685)		0.670 (0.656–0.684)	

CSS	Nomogram	0.774 (0.766–0.782)	<0.001	0.783 (0.770–0.797)	<0.001
	7th TNM stage	0.690 (0.680–0.700)		0.683 (0.667–0.699)	

*Note.* OS, overall survival; CSS, cancer-specific survival.

**Table 4:** Comparison of AUC between nomogram and TNM stage system in patients with EAC.

Survival		2-year survival AUC (TC)	5-year survival AUC (TC)	2-year survival AUC (IVC)	5-year survival AUC (IVC)
OS	Nomogram	0.834	0.853	0.844	0.866
	7th TNM stage	0.760	0.785	0.744	0.798

CSS	Nomogram	0.844	0.866	0.853	0.873
	7th TNM stage	0.772	0.801	0.754	0.808

*Note.* OS, overall survival; CSS, cancer-specific survival; TC, training cohort; IVC, internal validation cohort.

## Data Availability

Publicly available datasets were analyzed in this study. These data are available in Surveillance, Epidemiology, and End Results (SEER) database (https://seer.cancer.gov/). The datasets generated in this study are available from the corresponding author upon request.

## Additional Points

All the statistical analyses were performed using R version 3.4.2 software (the R Foundation for Statistical Computing, Vienna, Austria. http://http://www.r-project.org).

## Supplementary Materials

Supplementary Materials Supplemental Table 1: counts of different treatment types.
