# Supplementary material for: Predictive Model for Overall Survival and Cancer-Specific Survival in Patients with Esophageal Adenocarcinoma
Source: J Oncol. 2021 Sep 14;2021:4138575. doi: 10.1155/2021/4138575 (PMC8457966; doi:10.1155/2021/4138575)
Supplement: Supplementary Materials — Supplemental Table 1: counts of different treatment types. [file 4138575.f1.docx]

Supplemental Table 1 Counts of different treatment types

|  |  |  |  |  |  |  |  | Total |
| --- | --- | --- | --- | --- | --- | --- | --- | --- |
| Surgery | + | + | + | - | - | - | + | 2237 |
| Chemotherapy | + | + | - | + | - | + | - | 4545 |
| Radiation | + | - | + | + | + | - | - | 3787 |
| Total | 1461 | **157** | **21** | 1918 | 387 | 1009 | 598 |  |

+: positive; -: negative
